# The attitude of non-psychiatry doctors to psychiatry and its correlates in Mysore, South India

**DOI:** 10.1186/1753-6561-6-S4-P14

**Published:** 2012-07-09

**Authors:** S Faizan, BN Raveesh, V Anjali, R Lakshmanagowda Sujatha, K Sharath

**Affiliations:** 1 Department of Psychiatry, Mysore Medical College and Research Institute (MMC&RI), India; 2 Mysore Medical College and Research Institute (MMC&RI), India

## Introduction

The attitude of Non Psychiatry Doctors (NPD's) and Non psychiatry Post Graduate Residents (NPPG's) towards Psychiatry is crucially important because of its influence on impressionable medical students and the large number of psychiatric patients who present to Non Psychiatry Doctors like General Practitioners. Since the data on the attitude of NPD's and NPPG's to psychiatry in India is sparse we carried out this survey in a Tertiary Government teaching Hospital in Mysore, South India.

## Methods

A cross sectional survey of a sample consisting of 50 consenting Non Psychiatry Doctors (NPD's) and 85 Non Psychiatry Post Graduate Residents (NPPG's) obtained after proportional stratified random sampling based on specialty was carried out using a Pre Piloted modified version of the Attitude Toward Psychiatry (ATP-30) scale. Additional data related to their experience of Psychiatry as a discipline were recorded. The data collected were analyzed using the Chi square test and t test.

## Results

The response rate of NPD's was 72% (*n* = 36 from *N* = 50) and that for NPPG's was 76.4% (*n* = 65 from *N* = 85). The mean score of the Modified ATP scale used was 64.19 for NPD's and 61.76 for NPPG's (neutral attitude score 50). NPD's and NPPG's who believed in the importance of psychiatry in the curriculum referred more patients for psychiatric consultations (*P*<0.05) ,more NPD's than NPPG's felt that Psychiatry helps in the development of meaningful relationships with patients (*P*<0.05). More NPD's than NPPG's (*P*<0.05), among NPD's more recent graduates(P<0.005) and those who were younger (*P*<0.05) and with fewer years of practice were much more likely to believe that psychiatry has advanced considerably in recent years(*P*<0.05). Asked what would help them most in better detecting Psychiatric patients among patients presenting to them most NPD's reported more time in consultations (86.11%) while most NPPG's reported more appropriate interview techniques (76.92%).

## Conclusions

Non Psychiatry Doctors (NPD's) and Non Psychiatry Postgraduates (NPPG's) at a tertiary hospital in Mysore have a moderately positive attitude to Psychiatry. NPD's have a slightly more positive total attitude to Psychiatry as compared with NPPG's. NPPG's, while having a positive general attitude to psychiatry are more skeptical of recent advances in psychiatry and its importance as a discipline than their older mentors. This highlights the urgent need for better psychiatric education and more effective communication of the latest psychiatric knowledge to the younger generation of doctors.

